# GRP78 Impairs Production of Lipopolysaccharide-Induced Cytokines by Interaction with CD14

**DOI:** 10.3389/fimmu.2017.00579

**Published:** 2017-05-23

**Authors:** Kai Qin, Simin Ma, Heli Li, Min Wu, Yuanli Sun, Mingpeng Fu, Zilong Guo, Huifen Zhu, Feili Gong, Ping Lei, Guanxin Shen

**Affiliations:** ^1^Department of Immunology, School of Basic Medicine, Tongji Medical College, Huazhong University of Science and Technology, Wuhan, China; ^2^Institute of Integrated Traditional Chinese and Western Medicine, Tongji Hospital, Tongji Medical College, Huazhong University of Science and Technology, Wuhan, China

**Keywords:** GRP78, lipopolysaccharide, toll-like receptor 4, CD14, endocytosis

## Abstract

The 78-kDa glucose-regulated protein (GRP78) is a stress-inducible chaperone that resides primarily in the endoplasmic reticulum. GRP78 has been described to be released at times of cellular stress and as having extracellular properties that are anti-inflammatory or favor the resolution of inflammation. In the current study, we confirmed that GRP78 impaired the production of lipopolysaccharide-induced pro-inflammatory cytokines in GRP78-treated bone-marrow-derived dendritic cells (DCs). To explore the underlying mechanism, first of all, GRP78 was checked to be bound to the plasma membrane. Interestingly, such binding promoted endocytosis of toll-like receptor (TLR) 4 and reduction in TLR4 on the plasma surface had a key role in desensitization of GRP78-treated DCs to lipopolysaccharide. Given that cluster of differentiation (CD)14 is a crucial regulator of TLR4 endocytosis, interaction of GRP78 with CD14 was investigated next. Data showed that GRP78 co-localized with CD14 on the plasma membrane and glutathione-*S*-transferase-GRP78 precipitated CD14. In CD14 knockout mice, down-regulation of tumor necrosis factor-α and reduction in TLR4 on the plasma surface were abrogated in GRP78-treated DCs. Overall, these data suggested that GRP78 mediates endocytosis of TLR4 by targeting CD14 to favor the resolution of inflammation.

## Introduction

The 78-kDa glucose-regulated protein (GRP78), also referred to as immunoglobulin-binding protein (BiP), is a constitutively expressed resident protein of the endoplasmic reticulum (ER) present in all eukaryotic cells and belongs to the highly conserved heat shock 70 kDa protein (HSP70) family (1). GRP78 was initially identified as an “ER molecular chaperone” which protects cells against stress-induced apoptosis as a stress-inducible protein (2, 3). Our previous work has shown that GRP78 overexpression can protect insulinoma NIT-1 cells from cytotoxic T-cell-mediated lysis (4) and enhance survival of CHO cells in response to serum deprivation and oxidative stress (5).

Upregulation of GRP78 is induced by ER stress, leading to its cell surface expression and secretion into the extracellular compartment (6). Cell surface GRP78 forms complexes with a variety of cell-surface-anchored proteins (e.g., Cripto and T-cadherin) and extracellular ligands (e.g., activated α2-macroglobulin, Kringle 5, and Par-4) in tumor and endothelial cells leading to pro-survival or pro-apoptotic pathways (1, 7). However, secreted GRP78 exhibits immunoregulatory functions (8, 9). For instance, GRP78 treatment has been shown to induce dendritic cells (DCs) to express high levels of intracellular indoleamine 2,3-dioxygenase and to reduce expression of human leukocyte antigen (HLA)-DR and cluster of differentiation (CD)86. Besides, the phenotype of these anti-inflammatory DCs was stable regardless of exposure to lipopolysaccharide (LPS) (10). We also reported that GRP78 can increase the frequency of regulatory B-cells that produce interleukin (IL)-10 and highly express programmed death-ligand 1 and Fas ligand, resulting in the suppression of T-cell proliferation (11). In addition, GRP78 can induce myeloid antigen-presenting cells to maintain a “tolerogenic signature” upon LPS stimulation (12). For its potent immunomodulatory properties, GRP78 has been defined as one of the resolution-associated molecular patterns (RAMPs) (9).

Resolution-associated molecular patterns help to counterbalance the inflammatory effects of pathogen-associated molecular patterns (PAMPs) and damage-associated molecular patterns (DAMPs) to maintain immune homeostasis (9). Much more is known about how the immune system “senses” PAMPs and DAMPs than how it recognizes RAMPs. Detection of PAMPs is achieved through germline-encoded pattern recognition receptors (PRRs) for the initiation of innate immunity (13, 14). PRRs are a diverse family of structurally unrelated proteins that are grouped functionally by their ability to detect products (15). PRRs also recognize self-encoded DAMPs to contribute to the inflammatory response to dead cells (16, 17). The existence of self-derived PRR ligands has broadened the understanding of PRRs as determinants of the innate immune response. PAMPs and DAMPs have adjuvant and inflammatory properties, so it is rational for them to work through identical receptors. However, it is not clear if PRRs can sense the release of the stress-inducible RAMPs.

Central to our understanding of PRR biology is the toll-like receptor (TLR) family which, depending on the family member, resides at plasma or endosomal membranes. TLRs play an important part in host defense against pathogens, functioning as major sensors of self/non-self products (13, 14, 18). TLR4 signaling has a crucial role in host defense against Gram-negative bacteria by recognizing the outer membrane component, LPS (12). Upon treatment of cells with HSP70, TLR4 has been reported to transduce a signal depending on myeloid differentiation primary response gene 88 (MyD88) and require the presence of a co-receptor, either CD14 or myeloid differentiation factor (MD)-2 (19, 20). As a member of the HSP70 family, whether GRP78 can be recognized by TLR4 needs to be elucidated.

In the current study, we observed the immunomodulatory properties of GRP78 for its negative regulation to pro-inflammatory cytokines in LPS-induced bone marrow-derived dendritic cells (BMDCs). We found that cells treated with GRP78 down-regulated the expression of cell surface TLR4 by enhanced endocytosis. This endocytic event was promoted by GRP78 interacting with CD14 on the cell membrane, and was a key mechanism of desensitization of GRP78-treated DCs to LPS.

These discoveries establish that GRP78-induced TLR4 internalization is one of the mechanisms of its inflammatory resolution. Furthermore, the interaction between GRP78 and CD14 suggests that CD14 has a dual function in TLR4-mediated inflammation: a pro-inflammatory function induced by LPS and an anti-inflammatory function induced by GRP78.

## Materials and Methods

### Preparation of Recombinant Mouse GRP78

Recombinant mouse GRP78 was prepared as described in our previous report (11). Briefly, a plasmid encoding the full length of mouse GRP78 was transformed into *Escherichia coli* BL21 to generate glutathione-*S*-transferase (GST)-GRP78. Fusion protein was purified using Pierce^®^ Glutathione Spin Columns (16105; Thermo Scientific, Waltham, MA, USA). GRP78 was obtained by thrombin cleavage and identified by sodium dodecyl sulfate-polyacrylamide gel electrophoresis (SDS-PAGE) and immunoblotting. Protein concentration was detected using a Bicinchoninic Acid Protein Assay kit (Beyotime, China). Endotoxins were removed by a Pierce High-capacity Endotoxin Removal Resin (88274; Thermo Scientific), and the final endotoxin concentration of protein samples was <10 EU/mg. Negative control (NC) extracts from empty vector-transformed *E. coli* BL21 were prepared in the same way.

### Animals

C57BL/6 mice (HFK Bioscience, Beijing, China), C57BL/6 background TLR4 knockout (KO) mice (kindly donated by Professor Timothy R. Billiar, University of Pittsburgh, Pittsburgh, PA, USA), and CD14KO mice (The Jackson Laboratory, Bar Harbor, ME, USA) were bred in a specific pathogen-free facility and female mice were utilized at 6–8 weeks of age.

### Cell Culture

Bone marrow-derived dendritic cells from mice were generated as described previously (21). Cells were harvested after 7 days of culture with granulocyte-macrophage colony-stimulating factor (GM-CSF; 10 ng/mL; PeproTech, Rocky Hill, NJ, USA) and IL-4 (10 ng/mL; PeproTech). Bone-marrow-derived macrophages (BMDMs) were harvested after 7 days of culture with macrophage CSF (M-CSF; 50 ng/mL; PeproTech). HEK293T and DC2.4 cell lines were cultured in RPMI 1640 medium containing 10% fetal bovine serum.

### Reagents

Lipopolysaccharides (0111:B4) was obtained from Sigma-Aldrich (St. Louis, MO, USA). Antibodies specific for GST (sc-459) and β-actin (sc-47778) were purchased from Santa Cruz Biotechnology (Santa Cruz, CA, USA). Antibodies specific for GRP78 (ab32618), CD14 (ab182032), and TLR4 (ab22048) were obtained from Abcam (Cambridge, UK). Antibodies specific for interferon regulatory transcription factor (IRF)3 (4302) and IRF3 phosphorylated at Ser396(4947) were obtained from Cell Signaling Technology (Danvers, MA, USA). F(ab′)2-goat anti-mouse Alexa Fluor^®^555 (AF555) was purchased from Thermo Fisher Scientific. Anti-TLR4-PE was obtained from PharMingen (Becton Dickinson, Franklin Lakes, NJ, USA). Anti-LAMP1-PE, anti-CD14-PECy7, and permeabilization buffer were obtained from eBioscience (San Diego, CA, USA).

### Sequences and the Construction and Transfection of Plasmids

Mouse CD14 cDNA was synthesized by Brookline Scientific (Shanghai, China) and cloned into the pcDNA3.1 vector (Invitrogen, Carlsbad, CA, USA). For transient transfection of plasmids into 293T cells, jetPRIME reagents (Polyplus-transfection^®^, Illkirch-Graffenstaden, France) were used according to the manufacturer’s recommendations.

### Protein Labeling and Fluorescence Analyses

The labeling and purifying of Alexa Fluor^®^ 488-conjugated GRP78 (AF488-GRP78) was performed using a Protein Labeling kit (A10235; Molecular Probes, Eugene, OR, USA) according to the manufacturer’s recommendations, and AF488-BSA was used as an NC.

For flow cytometry (FCM) and immunofluorescence analyses, after blockade with 10% normal goat serum at 37°C for 30 min, cells were incubated with protein or antibodies at an appropriate temperature and time. Then, cells were detected by a flow cytometer (LSR II; BD Biosciences, Franklin Lakes, NJ, USA) or stained with Hoechst 33342 for cell nuclei before images were examined using a confocal laser scanning microscope (CLSM) (FV1000; Olympus, Tokyo, Japan). Images were analyzed by aFV10-ASW 2.1 Viewer (Olympus). The percentage of mean fluorescence intensity (MFI) for surface TLR4 was calculated using the following equation:

% of surface TLR4 (MFI)=(GRP78 treatment−isotype)/(NC−isotype)

### GST Pull-Down Assay

Membrane protein lysates from DC2.4 cells were extracted using a Membrane Protein Extraction kit (89842; Thermo Scientific). The cDNA encoding mouse GRP78 and fragments of GRP78 were cloned into the pGEX-4T3 vector. Recombinant fusion proteins of GST-GRP78, GST fragments of GRP78, and GST control (20 µg) were incubated with 500 µL DC2.4 cell membrane protein lysates (1 × 10^6^ cells) for 1 h on ice, followed by the addition of 50 µL glutathione agarose beads (16105; Thermo Scientific) for incubation overnight at 4°C with constant mixing. Protein complexes were washed extensively with buffers. The precipitates were analyzed by 10% SDS-PAGE and blotted using antibody specific for GST, GRP78, TLR4, and CD14.

### Proteins and the Protein–Protein Interactive Precipitation Assay

The physical interaction between GRP78 and CD14 was determined by the protein–protein interactive precipitation assay. The cDNA encoding mouse CD14 was cloned into the pOptiVEC-hIgG1-Fc vector. Recombinant CD14-Fc and Fc control proteins were purified by protein G-agarose (sc-2002; Santa Cruz Biotechnology) from culture supernatants of HEK293T cells transfected transiently with relative plasmids, followed by incubation with GRP78 at 4°C overnight. Precipitates were analyzed by 10% SDS-PAGE and blotted using antibody specific for hIgG1-Fc, GRP78, and CD14.

### Enzyme-Linked Immunosorbent Assay (ELISA)

Concentrations of IL-6, tumor necrosis factor (TNF)-α, and interferon (IFN)-β in culture supernatants were determined by ELISA kits from BioLegend (San Diego, CA, USA) following the manufacturer’s recommendations.

### RNA Quantification

Cell total RNA was extracted with TriZol^®^Reagent (Invitrogen) and cDNA was generated using a HiFiScript^®^ cDNA Synthesis kit (CW Biotech, Beijing, China). Quantitative real-time polymerase chain reaction (qRT-PCR) analyses were carried out using an SYBR Green Real-time PCR kit (Toyobo, Osaka, Japan) in a LightCycler^®^ (Bio-Rad Laboratories, Hercules, CA, USA). Data were normalized to glyceraldehyde 3-phosphate dehydrogenase, and fold changes were analyzed using the formula: 2^−Δ Δ Ct^.

### Statistical Analyses

Mean values were compared using the Student’s *t*-test (two groups) or one-way ANOVA (three or more groups). Results are the mean and standard deviation (SD). All ELISA, qPCR, and fluorescence-activated cell sorting (FACS) experiments were performed thrice, and one representative result was presented. The immunofluorescence experiments and protein blots shown are representative data from at least three independent experiments.

## Results

### GRP78 Inhibits Production of the Cytokines Induced by LPS in BMDCs and BMDMs

**Figure 1:**
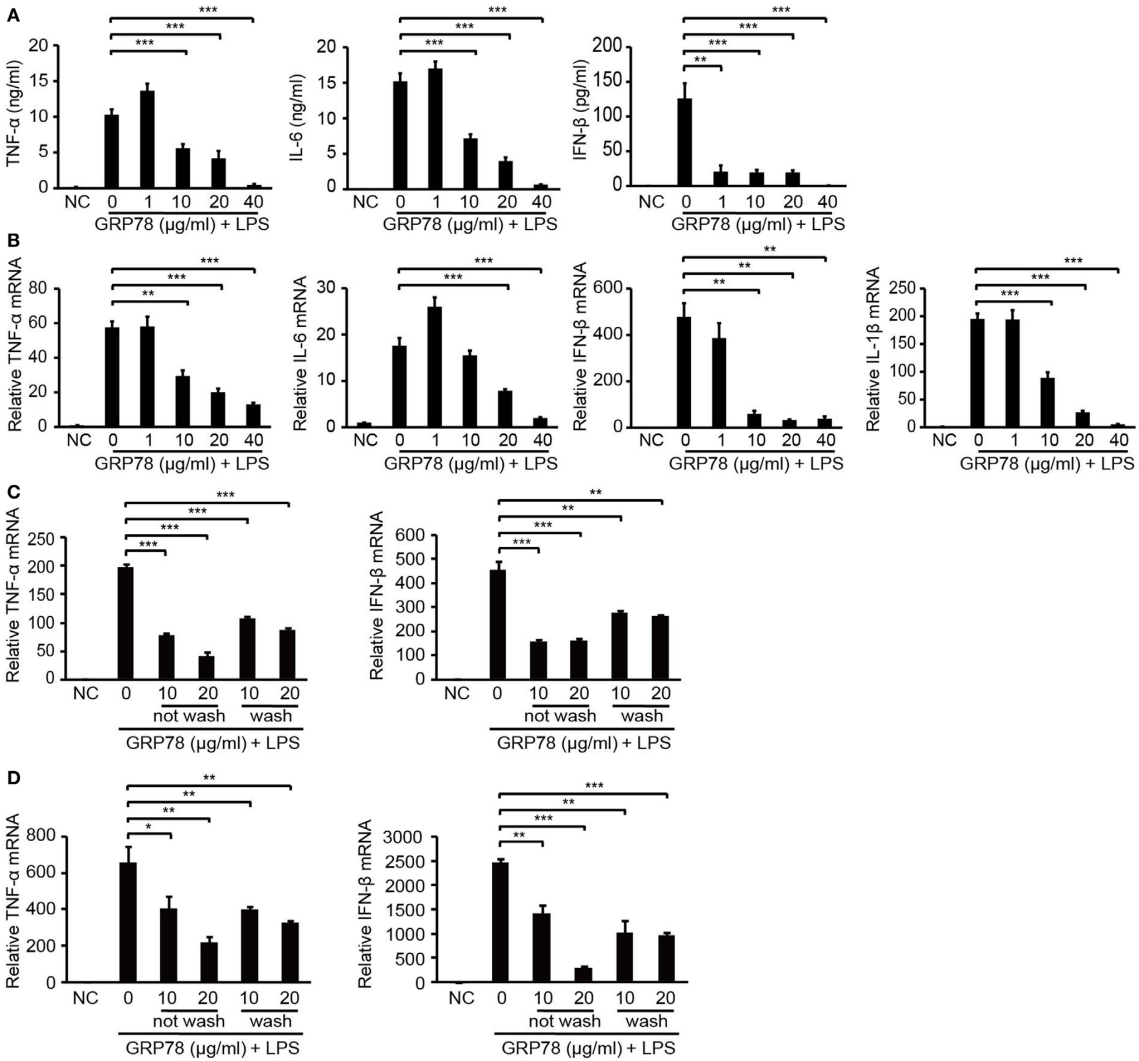
**GRP78 inhibits production of lipopolysaccharide (LPS)-induced cytokines in bone marrow-derived dendritic cells (BMDCs) and bone marrow-derived macrophages (BMDMs)**. Enzyme-linked immunosorbent assay **(A)** and real-time quantitative polymerase chain reaction (qRT-PCR) **(B)** of tumor necrosis factor (TNF)-α, interleukin (IL)-1β, IL-6, and interferon (IFN)-β in murine BMDCs unstimulated (NC) or stimulated for 4 h **(A)** or 2 h **(B)** by LPS (100 ng/mL) and simultaneously by GRP78 at the concentrations indicated. qRT-PCR of TNF-α and IFN-β in BMDCs **(C)** or BMDMs **(D)** which were treated with GRP78 (10 or 20 µg/mL) for 2 h, then were washed thrice with culture medium (wash) or not (not wash), followed by 100 ng/mL LPS for 2 h. Error bars represent mean ± SD from triplicate samples in one experiment. *P < 0.05, **P < 0.01, and ***P < 0.001.

To confirm the immunomodulatory properties of GRP78, the production of inflammatory cytokines in GRP78-treated BMDCs was measured. LPS stimulated BMDCs to release large quantities of pro-inflammatory cytokines such as TNF-α, IL-6, and IFN-β. However, such promotion could be attenuated by simultaneous supplementation of GRP78 in a concentration-dependent manner (Figure 1A). Even as little as 1 µg/mL of GRP78 could reduce IFN-β production significantly (*P* < 0.01). GRP78 (40 µg/mL) led to nearly complete abolishment of the expression of those cytokines. qRT-PCR results corroborated the data given earlier (Figure 1B). To exclude the possibility that GRP78 impaired LPS-induced cytokine expression by inhibiting the interaction between LPS and its receptor, instead of simultaneous treatment with LPS and GRP78, cells were treated with GRP78 first, and then rinsed extensively with culture medium (wash) or not (not wash) and, finally, stimulated with LPS. Expression of TNF-α and IFN-β in both groups was relatively lower than those in cells stimulated only by LPS (Figure 1C). A similar anti-inflammatory effect of GRP78 was observed in LPS-stimulated BMDMs (Figure 1D).

### GRP78 Is Bound, but Not Endocytosed, by DCs

Modulatory effects of extracellular HSP70 members on innate immunity have been heavily debated, as neither the immunomodulatory receptors responsible for binding nor the intracellular signaling events have been established definitively (22, 23). To identify the functional mode of GRP78, we investigated whether GRP78 is bound by cell surface receptors or transported into cells.

**Figure 2:**
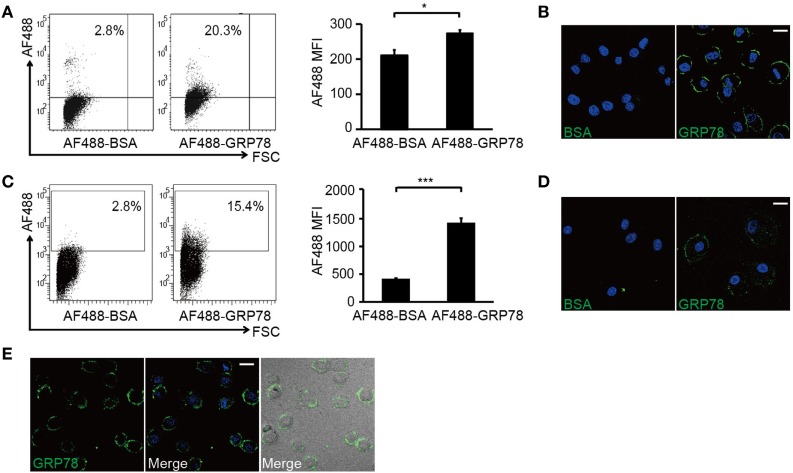
**GRP78 is bound (4°C) but not endocytosed (37°C) by dendritic cells**. DC2.4 cells **(A,B)** or bone marrow-derived dendritic cells **(C,D)** were incubated with AF488-GRP78 or BSA (1 µM) at 4°C for 1 h. Fluorescence was detected by flow cytometry (FCM) **(A,C)** or confocal laser scanning microscopy (CLSM) **(B,D)**. **(E)** DC2.4 cells were stained with AF488-GRP78 or BSA at 37°C for 1 h. The location of GRP78 was detected by CLSM. Error bars represent mean ± SD from triplicate samples in one experiment. *P < 0.05 and ***P < 0.001. All images for all panels are representative of at least three independent experiments in which >100 cells were examined and >95% of cells showed similar staining. Scale bar, 20 µm.

Flow cytometry analyses showed that DC2.4 cells and BMDCs were stained by AF488-GRP78 but not AF488-BSA (Figures 2A,C). CLSM revealed that, at 4°C (to block vesicular trafficking), 90–95% of AF488-GRP78-stained cells emitted green fluorescence, whereas cells treated with AF488-BSA were not stained. The fluorescence of AF488 was localized only at the plasma membrane (Figures 2B,D). Moreover, when such staining was conducted at 37°C, fluorescence did not become “grainy” and, thereafter, no part of the grains were clustered (Figure 2E).

Collectively, these results suggested that it was through binding with cell-surface structures, not by being endocytosed, did GRP78 exert its modulatory effects.

### GRP78 Reduces Cell Surface Expression of TLR4 on DCs

TLR4 is a crucial “switch” for mediating LPS-induced signal transduction (24). In addition, TLR4-dependent pathways ultimately lead to the transcription of genes encoding various pro-inflammatory mediators (e.g., TNF, IL-6, and IL-1β) (18, 25, 26). After confirming that GRP78 could bind with unknown cell surface structures on DCs, we examined whether GRP78 could interact with TLR4 and, if so, its role in TLR4-triggered responses.

**Figure 3:**
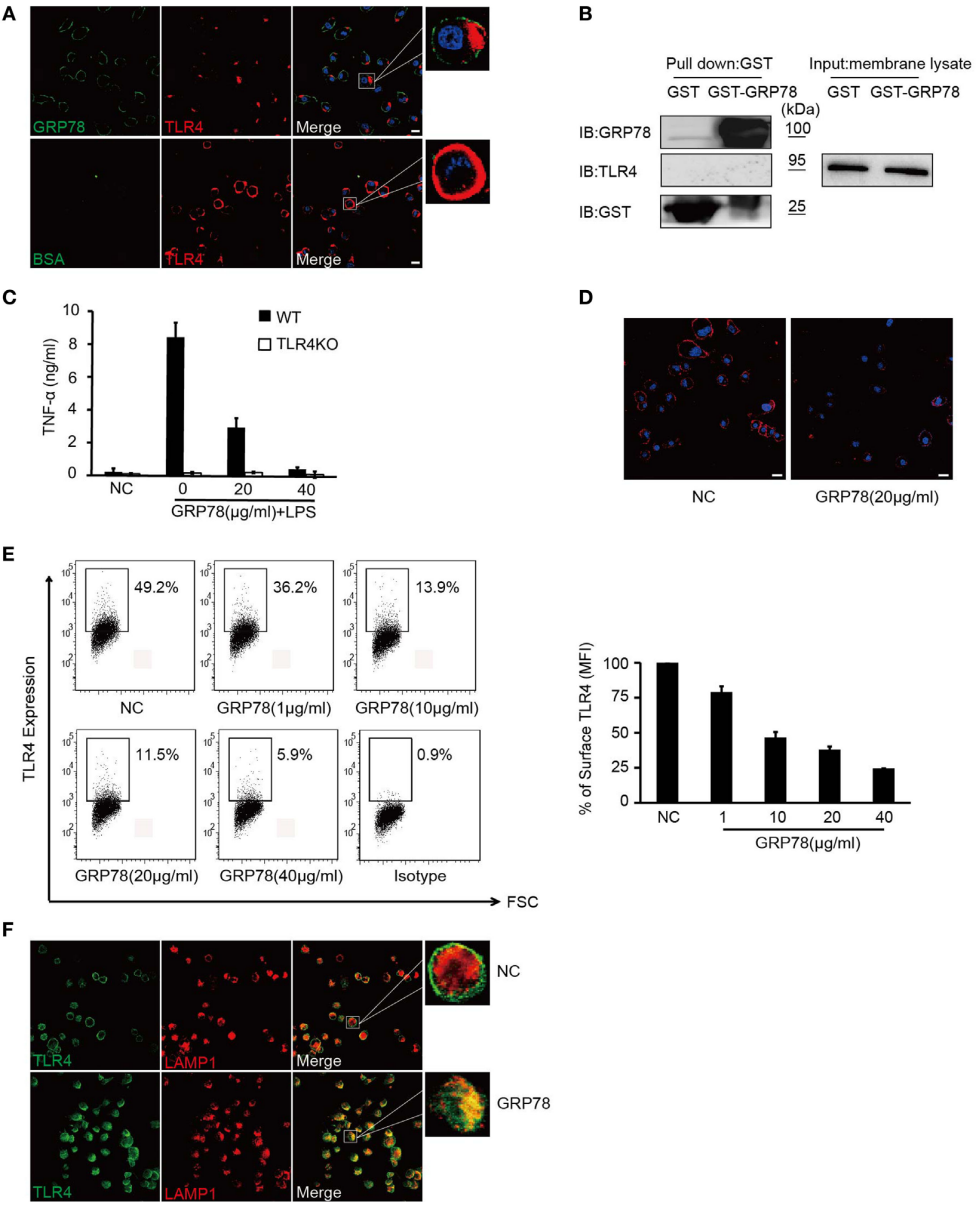
**GRP78 down-regulates surface levels of TLR4 on dendritic cells**. **(A)** DC2.4 cells were treated with AF488-GRP78 or BSA (1 µM) at 4°C for 1 h, and then TLR4 was stained using anti-TLR4 and AF555-conjugated (red) goat anti-mouse IgG. **(B)** Immunoblot analyses of the interaction of TLR4 with GST-GRP78, after incubation with membrane lysates of DC2.4 cells, followed by precipitation by glutathione agarose beads. **(C)** Enzyme-linked immunosorbent assay of TNF-α in wild-type or TLR4KO bone marrow-derived dendritic cells stimulated for 4 h by LPS together with GRP78 at the concentrations indicated. **(D)** DC2.4 cells were incubated with GRP78 at 37°C for 30 min, surface TLR4 was stained and detected by confocal laser scanning microscopy (CLSM). **(E)** DC2.4 cells were treated with GRP78 for 30 min. TLR4 surface staining was measured by FCM. Dot plots of FCM data (left) and percentage of mean fluorescence intensity (MFI, right) are depicted. Percentage of surface TLR4 (MFI) were analyzed using the following formula: (GRP78 treatment − isotype)/(NC − isotype). **(F)** DC2.4 cells were incubated with GRP78 at 37°C for 1 h, surface TLR4 (green) on non-permeabilized cells and intracellular TLR4 and LAPM1 (red) on permeabilized cells were stained and detected by CLSM. Error bars represent mean ± SD from triplicate samples in one experiment. All images for all panels are representative of at least three independent experiments in which >100 cells were examined and >95% of cells showed similar staining. Scale bar, 20 µm.

Dendritic cells were stained with GRP78 (green) for 30 min and then stained with TLR4 (red). Interestingly, as DCs were stained with GRP78 green fluorescence at the plasma membrane, TLR4 red fluorescence was localized beneath plasma, whereas in AF488-BSA-stained control cells, it was distributed evenly at the plasma membrane. Orange-to-yellow overlap fluorescence could not be observed in merged images (Figure 3A), so GRP78 and TLR4 were not co-localized. Furthermore, in GST precipitation assays, GST-GRP78 failed to precipitate TLR4 (Figure 3B). Therefore, GRP78 probably interacted not with TLR4, but with other partners.

Although GRP78 did not interact directly with TLR4, it did have roles in TLR4-triggered responses, because GRP78 showed dose-dependent inhibition to TNF-α in LPS-induced wild-type (WT) BMDCs (Figures 1 and 3C). Approximately 40 µg/mL GRP78 treatment nearly abrogated TNF-α production, which was in close proximity with no response in TLR4 KO BMDCs (Figure 3C). Hence, how did GRP78 attenuate TLR4-mediated responses?

Various negative regulatory mechanisms have evolved to attenuate the signaling of TLRs to sustain immunologic balance, including the reduction of TLR expression (18). It is believed that the endocytosis of plasma membrane-localized TLRs down-regulates their signaling functions after a microbial encounter for signal termination (27). To ascertain whether GRP78 could reduce cell surface expression of TLR4, the intensity of TLR4 on DCs before and after GRP78 treatment was assessed.

Confocal laser scanning microscopy (Figure 3D) showed that evenly distributed TLR4 fluorescence was reduced after GRP78 treatment. Using the loss of cell surface expression as a readout for TLR4 endocytosis (28), we observed that the MFI of cell surface TLR4 decreased dramatically as the GRP78 concentration increased (Figure 3E). This FACS-based assay was consistent with the anti-inflammatory functions of GRP78 (Figure 1). By counterstaining cells with an antibody against TLR4 and the late endosomal/lysosomal marker protein, LAMP-1, we observed a decrease in the expression of plasma membrane TLR4 and an increase in co-localization between intracellular TLR4 and LAPM-1 during 1 h of incubation with GRP78 (Figure 3F). We speculated that TLR4 was degraded in lysosomes after GRP78 stimulation.

Collectively, these data suggested that GRP78 could inhibit TLR4 signaling through the promotion of TLR4 endocytosis and reduction of its expression on the surface of DCs.

### GRP78 Interacts with CD14

Although the GST precipitation assay did not support the notion of direct interaction of GRP78 with TLR4, GRP78 did reduce the cell surface expression of TLR4 to counteract LPS-induced production of pro-inflammatory cytokines. Given that CD14 is a crucial regulator of TLR4 endocytosis and is known to chaperone LPS molecules to TLR4 signaling complexes (28, 29), we tested whether GRP78 could interact with CD14 to regulate TLR4 endocytosis.

**Figure 4:**
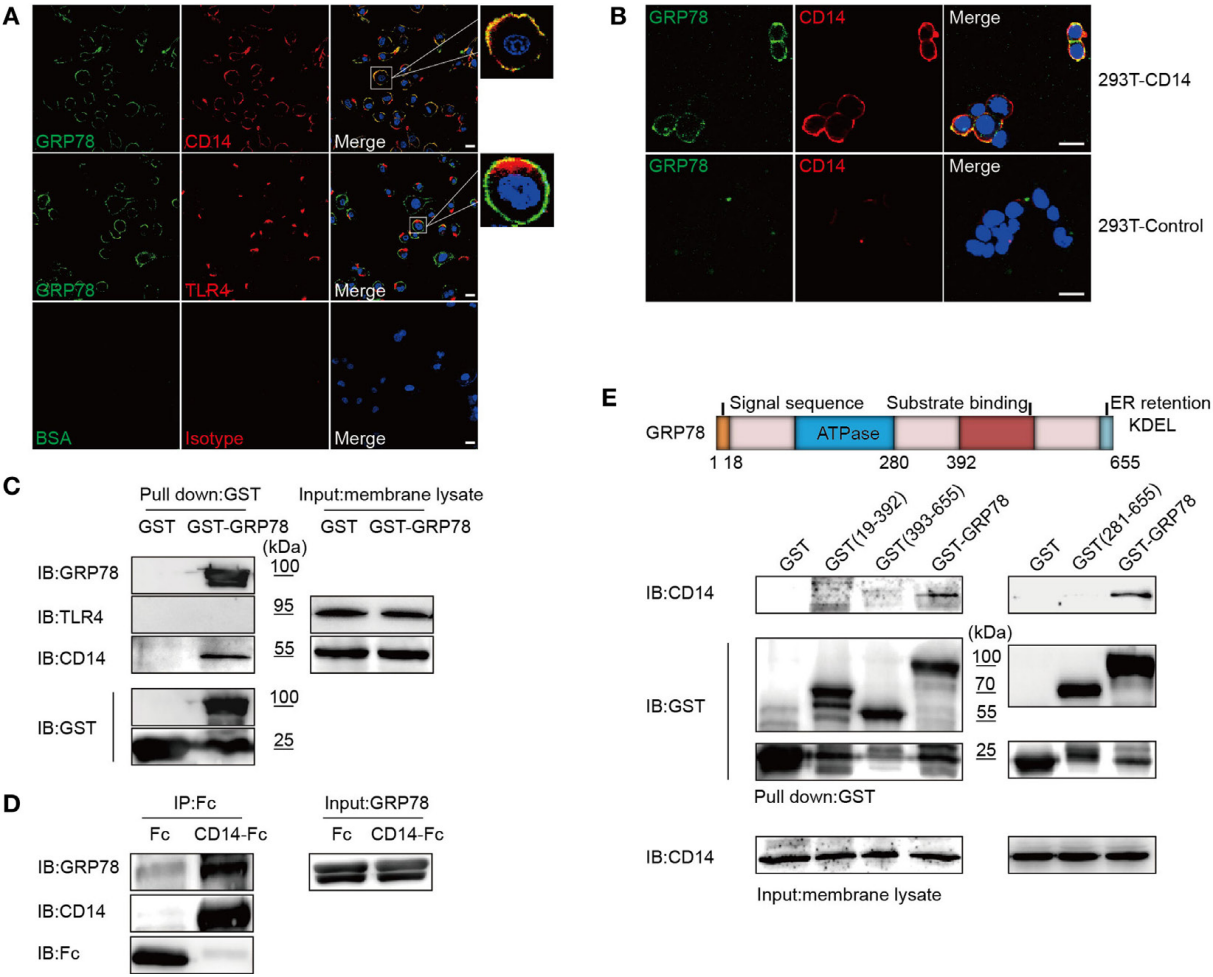
**GRP78 Interacts with CD14**. **(A)** DC2.4 cells were treated with AF488-GRP78 (green) for 1 h at 4°C, and then TLR4 and CD14 (red) were stained. **(B)** pcDNA3.1-CD14 transiently transfected HEK293T cells were incubated with AF488-GRP78 and then CD14 was stained. Fluorescence localization was determined by confocal laser scanning microscopy. **(C)** Immunoblotting of the interaction of proteins with GST-GRP78, after incubation with membrane lysates of DC2.4 cells, followed by precipitation by glutathione agarose beads. **(D)** Protein precipitation study of CD14-Fc with GRP78. The cDNA encoding mouse CD14 was cloned into the pOptiVEC-hIgG1-Fc vector. Recombinant CD14-Fc and Fc control proteins from culture supernatants of HEK293T cells transiently transfected with plasmids were immobilized onto protein G-agarose, followed by incubation with GRP78 at 4°C overnight. The precipitates were immunoblotted using antibody specific for hIgG1-Fc, GRP78, and CD14. **(E)** Schematic representation of recombinant GRP78 (upper). Interaction of GRP78 fragments (amino acids in parentheses) with CD14 was assessed by GST precipitation, as described in **(D)**. All images for all panels are representative of at least three independent experiments in which > 100 cells were examined and > 95% of cells showed similar staining. Scale bar, 20 µm.

Unlike no co-localization of GRP78 and TLR4, double immunofluorescence staining showed that the pattern of staining of DC2.4 cells with CD14 red fluorescence reflected a high degree of co-localization with that of GRP78 green fluorescence (Figure 4A). Furthermore, GRP78 was found to stain the plasma membrane of 293T cells transfected with CD14, but failed to stain the 293T mock control (Figure 4B). Orange-to-yellow overlap fluorescence in merged images suggested the co-localization of GRP78 and CD14 on the plasma membrane. To ascertain whether GRP78 binds to CD14 directly, the GST precipitation assay was carried out again. CD14 was precipitated *via* binding to GST-GRP78, whereas TLR4 was not (Figure 4C). Recombinant fusion protein CD14-Fc was also used to determine the ability of CD14 to bind to GRP78 by the protein–protein interactive precipitation assay. Recombinant GRP78 was precipitated *via* binding to CD14-Fc, but control Fc was not precipitated (Figure 4D).

GRP78 contains two important domains: an adenosine triphosphate (ATP)-binding domain and a substrate-binding domain. The former is required for ATPase catalytic activity and the latter is responsible for binding to the substrate protein (1). To ascertain which domain was required for interaction with CD14, GST fusion proteins containing various fragments of GRP78 were used to carry out the GST precipitation assay. Surprisingly, none of the truncated GRP78 interacted with CD14, and only full-length GRP78 retained the ability to bind to CD14 (Figure 4E).

Collectively, this series of experiments revealed that GRP78 interacted with CD14 using its complete conformation.

### Negative Regulation of GRP78 on the TLR4 Pathway Is Dependent on CD14

As a TLR4 accessory protein, CD14 functions not only in ligand transport but also in receptor transport to endosomes (28, 29). To determine the effect of binding of CD14 by GRP78 on TLR4 transport, BMDCs from WT and CD14KO mice were monitored for cytokine production and TLR4 endocytosis.

**Figure 5:**
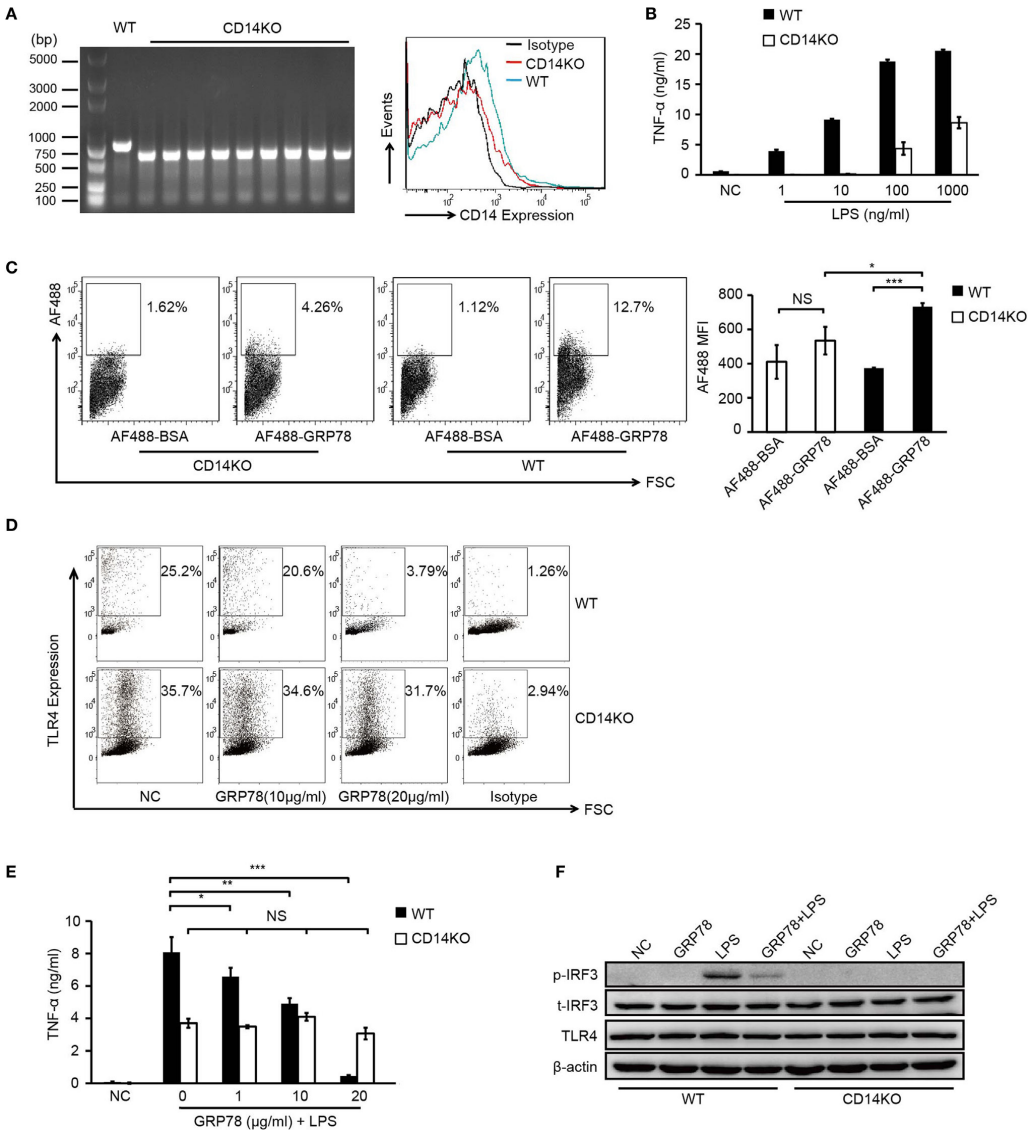
**GRP78-induced TLR4 endocytosis is CD14 dependent**. **(A)** CD14 levels on WT and CD14KO bone marrow-derived dendritic cells (BMDCs) were determined by PCR (left) and FCM (right). PCR products of CD14 gene in the genomic DNA from WT and CD14KO mouse: CD14 knockout = ~600 bp, wild type = 840 bp (according to the Jackson Laboratory). **(B)** Enzyme-linked immunosorbent assay (ELISA) of TNF-α in WT or CD14KO BMDCs stimulated for 4 h by LPS at the concentrations indicated. **(C)** FCM analyses for GRP78 binding with WT or CD14KO BMDCs. Representative dot plots (left) and MFI (right) are shown. **(D)** WT or CD14KO BMDCs were treated with 10 µg/mL or 20 µg/mL GRP78 for 30 min. Surface TLR4 was determined by FCM. **(E)** ELISA of TNF-α in WT or CD14KO BMDCs treated with LPS (100 ng/mL) and GRP78 for 4 h. **(F)** WT or CD14KO BMDCs were stimulated with GRP78 (20 µg/mL), LPS (100 ng/mL), or mixtures of GRP78 and LPS at 37°C for 30 min, cells were lysed, and phoepho-IRF3, total-IFR3, TLR4, and actin were detected by immunoblotting. Error bars represent mean ± SD from triplicate samples in one experiment. NS, not significant; *P < 0.05, **P < 0.01, and ***P < 0.001.

PCR identification of the genomic DNA and FCM analyses confirmed the CD14 deficiency in CD14KO mice (Figure 5A). As expected, in the absence of CD14, the concentrations of LPS needed to activate TNF-α production increased by several orders of magnitude (Figure 5B) and AF488-GRP78 showed no significant difference in staining BMDCs compared with the bovine serum albumin control. However, the percentage and MFI of AF488-GRP78^+^ cells from WT mice were much higher than those from CD14KO mice (Figure 5C). In subsequent experiments in CD14KO BMDCs, no alterations were found in surface expression of TLR4 which was down-regulated previously by GRP78 in WT BMDCs in a concentration-dependent manner (Figure 5D). Furthermore, CD14KO BMDCs exhibited no changes in TNF-α production upon treatment with GRP78 and LPS (Figure 5E). However, inflammatory endocytosis of TLR4 mediated by CD14 can activate TIR-domain-containing adapter-inducing interferon-β (TRIF)-IRF3 signaling following LPS stimulation (28), we found GRP78 did not induce IRF3 phosphorylation and inhibited the activation of IRF3 triggered by LPS (Figure 5F), suggesting that GRP78 induced the non-inflammatory endocytosis of TLR4.

Collectively, these results suggested that the immunomodulatory property of GRP78 in LPS-induced TLR4 signaling was dependent upon CD14.

## Discussion

Recent studies have suggested that GRP78 shows potent immunomodulatory properties in an extracellular form (8–11). GRP78 has been defined as an RAMP to counterbalance the inflammatory effects of PAMPs and DAMPs (9). Despite these advances, we lack a clear understanding how this extracellular protein is sensed by immune cells and the identity of the cell-surface receptor that endows GRP78 with immune functions that are powerfully immunomodulatory and pro-resolutory.

Extracellular HSP70 members have been reported to bind with the paired receptors Siglec-5 and Siglec-14 expressed on monocytes and neutrophils (30) as well as to engage scavenger receptors for uptake to mediate their intracellular responses (31), or to interact with CD14 expressed on the cell surface of immune tissues to exert pro-inflammatory responses (19, 32). Attempts have also been made to identify the GRP78-binding structures expressed on cell surfaces. Corrigall et al. reported that in the human immune system GRP78 has a specific, as yet unidentified receptor(s), that is expressed in myeloid lineage cells (33). Li et al. showed that secreted GRP78 could utilize cell surface GRP78 on colon cancer cells as its receptor to activate intracellular signaling for proliferation (34).

In this article, we observed that extracellular GRP78 showed a powerful effect on the down-regulation of the production of LPS-stimulated pro-inflammatory cytokines in DCs. Rapid reduction of the expression of surface TLR4 induced by GRP78 could account for these results.

TLR4 is a specific receptor for LPS (13). Its MyD88-mediated signaling occurs mainly at plasma membranes, which results in nuclear factor-kappa B (NF-κB) activation and induction of pro-inflammatory mediators such as TNF-α and IL-6. In contrast, TRIF-mediated signaling in response to LPS occurs at the endosomal membrane after internalization of the TLR4 that, in turn, activates IRF3, resulting in the production of IFN-β, IP-10, and other IRF3-dependent genes (35, 36). The expression and function of TLR4 can be down-regulated by anti-inflammatory cytokines, especially transforming growth factor (TGF)-β and IL-10 (18). In our experiments, the expression of TGF-β and IL-10 was barely detectable in DCs treated with LPS or GRP78 (data not shown). The reduction of TLR4 expression could also be achieved by degradation through ubiquitylation (18). After internalization induced by LPS, TLR4 is ubiquitinated and trafficked to endosomes/lysosomes for degradation, which creates an inducible TLR4 deficiency at the plasma membrane. TLR4 deficiency would desensitize the cell after LPS activation (27). In the current study, we provided evidence that GRP78 induced the internalization and translocation of TLR4 to lysosomes, and that lysosomal degradation of TLR4 resulted in inefficient activation of TLR4 signaling by LPS. The ability of GRP78 to create a TLR4 deficiency at the plasma membrane would be expected to render it a “TLR4 antagonist.”

CD14 was considered to be a marker of myeloid lineage cells at first (37). Subsequently, CD14 was identified as the co-receptor working with TLR4 and MD-2 (38–40) to combine with complexes of LPS and LPS BiPs (41). Upon the detection of LPS, CD14 delivers TLR4 to endosomes to induce an inflammatory endocytosis pathway (29). CD14 blockade can abrogate the production of MyD88- and TRIF-dependent cytokines such as TNF-α, IL-1, IL-6, and IFN-β (41). Heat shock protein family A member 1 (HSPA1), a member of the HSP70 family, has been shown to utilize TLR4 and TLR2 in driving the inflammatory response through a CD14-dependent pathway (19, 42). As a HSP70 family member, GRP78 shares 60% amino acid homology with HSPA1 (43). Our results supported the notion that GRP78 exerted its properties also through a CD14-dependent pathway. However, such interaction with CD14 was anti-inflammatory and pro-resolutory. Two possible explanations were that GRP78 restrains LPS-induced TLR4 signaling by blocking CD14, or GRP78 induces the endocytosis of TLR4. CD14 is not required for MyD88-dependent signaling at higher LPS concentrations (28, 44, 45) (in the current study, LPS was used at concentration as high as 100 ng/mL) and a correlation between GRP78 stimulation and down-regulation of expression of cell surface TLR4 was recorded in our experiments, so enhanced endocytosis of TLR4 was the major anti-inflammatory mechanism of GRP78. Furthermore, TLR4 never reappeared at the plasma membrane after endocytosis but CD14 reappeared later (29). This may explain why GRP78 could persistently suppress the production of pro-inflammatory cytokines induced by LPS.

The GRP78-induced anti-inflammatory endocytosis described in this article was different from the inflammatory endocytosis of TLR4 discussed traditionally, which activates TRIF-mediated signaling to promote subsequent expression of IFNs and IFN-stimulated genes, because the levels of IFNs were reduced in our experiments. This “endocytic heterogeneity” suggests that the endocytosis and signaling of TLR4 should be a dissociable process or there should be different means by which TLR4 can be delivered into endosomes. Zanoni et al. observed such heterogeneity when BMDMs and DCs differed in their ability to allow TLR4 and FcγR1 to enter phagosomes when using a common means of internalization (phagocytosis) (28). Zanoni et al. also recorded the non-inflammatory endocytosis that penta-acylated LPS from Rhodobacter sphaeroides (Rs-LPS) did not trigger myddosome formation, *IL-1b* and *Rsad2* expression while its hexa-acylated counterpart from *E. coli* (Ec-LPS) could elicit these responses, although Rs-LPS was capable of modest TLR4 endocytosis (29). Similar to GRP78, Rs-LPS could lead to a near complete loss of TRIF-dependent Rsad2 expression and MyD88-dependent Il1b expression induced by Ec-LPS (29). Rajaiah et al. (46) also reported that endotoxin-tolerized macrophages exhibited significant internalization of TLR4, but that IFN-β production was blocked completely. How CD14 mediates TLR4 endocytosis after stimulation of GRP78, and how CD14 recognizes different ligands to regulate the trafficking of TLR4 into the endosome, merit further investigation.

Recent studies have confirmed that CD14 has more functions than simply delivering ligands to TLRs, and that its biologic effects are different from TLRs signaling. Under stimulation of LPS, DCs induces the activation of CD14-dependent Src family kinases and phospholipase Cγ2, influx of extracellular Ca^2+^, and NF of activated T-cells translocation, which is necessary to cause the apoptotic death of terminally differentiated DCs (44). These observations suggest that CD14 is not only the accessory protein of TLRs signaling but also a PRR with immunomodulatory properties. Our study also suggested that CD14 had a dual role in TLR4 signaling: a pro-inflammatory role induced by LPS and an anti-inflammatory role induced by GRP78.

## Conclusion

Our work demonstrated that GRP78 suppresses LPS-induced production of cytokines by promoting TLR4 internalization, during which CD14 is a key receptor for GRP78. Importantly, we showed, for the first time, that GRP78 could be bound by CD14. The ability of GRP78 to arrest LPS-induced activation of TLR4 signaling was dependent upon CD14.

These discoveries suggest that GRP78 could act as a negative regulator and that CD14 could have a dual role in the innate immune response. Overall, our results identified the immunomodulatory mechanism of secreted GRP78 in LPS-induced inflammation, and shed new light on the therapy of endotoxin-based shock or other inflammatory conditions.

## Ethics Statement

This study was carried out in strict accordance with the recommendations of the Regulations for the Administration of Affairs Concerning Experimental Animals of the State Science and Technology Commission. The protocol was approved by the Ethical Review Board of Tongji Medical College.

## Author Contributions

KQ and PL designed the study, analyzed the data, and wrote the manuscript; KQ, SM, and HL carried out experiments and statistical evaluations; HL and MW undertook animal cultivation; YS, MF, ZG, and HZ carried out cell cultures; and GS, PL, and FG provided funding.

## Conflict of Interest Statement

The authors declare that the research was conducted in the absence of any commercial or financial relationships that could be construed as a potential conflict of interest.
